# Dual IRE1 targets: Determinants of the cell fate?

**DOI:** 10.1111/febs.70624

**Published:** 2026-06-15

**Authors:** Eva Billat, Léane Legrand, Tony Avril, Elodie Lafont

**Affiliations:** ^1^ INSERM UMR1242 Oncogenesis Stress Signaling; University of Rennes France; ^2^ Centre Eugène Marquis Rennes France

**Keywords:** cancer, CD95, dual IRE1 target, ER stress, IRE1, proteostasis, RIDD, UBE2D3, XBP1s

## Abstract

IRE1α (hereafter referred to as IRE1) is one of the sensors implicated in the unfolded protein response that controls the ER protein homeostasis (also known as proteostasis). Alteration of proteostasis is observed in many diseases, making IRE1 a central element of cell adaptability upon disease onset and progression. Upon ER stress, IRE1 initially promotes cell adaptation. Conversely, when proteostasis cannot be restored, IRE1 activation can lead to cell death. IRE1 activity mainly regulates two pathways: the formation of the transcription factor XBP1s; and the regulated IRE1‐dependent decay (RIDD) of RNA, which can contribute to both cell adaptation and death. Hence, on one hand, IRE1 favors gene expression, while on the other hand it induces transcript degradation. We have recently identified two genes, *CD95* and *UBE2D3*, which are targeted by both signaling branches downstream of IRE1 RNase's activity, resulting in a dual and opposing regulation of their expression. We propose naming these targets ‘DIT’ for Dual IRE1 Targets. Interestingly, other IRE1 targets, such as BiP and DGAT2, have previously been reported to be regulated by XBP1s and RIDD in separate studies. We hypothesize that regulation of DIT could be crucial to tilt the balance between the pro‐adaptative and pro‐death outcomes of IRE1, especially in pathological contexts. Therefore, understanding this regulation could be key to unraveling the IRE1/XBP1s/RIDD signaling network. Here, we explore these hypotheses by highlighting various aspects of the regulation of IRE1 branches, and reviewing the DIT identified in the literature so far.

## Graphical abstract

IRE1 is an ER stress sensor that restores protein homeostasis through two signaling activities: XBP1s, which upregulates its target gene expression or RIDD which downregulates its target transcripts. We recently identified Dual IRE1 Targets (DIT) which are modulated by both XBP1s and RIDD activities. We hypothesize that DIT are determinants of cell fate as their fine regulation by IRE1 shapes the cell stress response and interactions with its microenvironment.

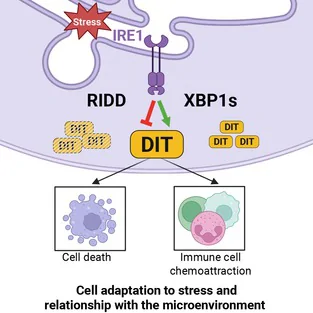

**Abbreviations**
ATF6α: activating transcription factor 6 alpha
BiP: binding immunoglobulin protein
CD95L: CD95 ligand
CHOP: C/EBP homologous protein
DGAT: diacylglycerol O‐acyltransferase
DIT: dual IRE1 targets
DN: dominant negative
DR: death receptor
eIF2α: eukaryotic initiation factor 2 α
ER: endoplasmic reticulum
FOXA3: forkhead box A3
GB: glioblastoma
GRP78: glucose‐related protein 78
IRE1: inositol‐requiring enzyme 1
IκBα: NF‐κB inhibitor alpha
NF‐κB: nuclear factor‐kappa B
PERK: protein kinase RNA‐like endoplasmic reticulum kinase
PUMA: p53 upregulation modulator of apoptosis
RIDD(LE): regulated‐IRE1‐dependent decay (lacking endomotif)
RNase: endoribonuclease
Rtcb: RNA 2′,3′‐cyclic phosphate and 5’‐OH ligase
TF: transcription factor
Tg: thapsigargin
TNBC: triple‐negative breast cancer
TNFR1: tumor necrosis factor receptor 1
TRAIL‐R1/R2: TNF‐related apoptosis‐inducing ligand receptor 1/2
UBE2D3: ubiquitin‐conjugating enzyme E2 D3
UPR: unfolded protein response
XBP1(s): X‐box binding protein 1 (spliced)

## Introduction

Protein homeostasis, also named proteostasis, consists in the balance of multiple molecular processes which lead to proper protein manufacturing, including protein synthesis, folding, modification, localization, and degradation. Alteration of proteostasis is observed in many diseases such as neurodegenerative, metabolic, muscular, immune, and developmental diseases as well as cancer [1, 2]. The endoplasmic reticulum (ER) is a central organelle for protein homeostasis as many proteins enter its lumen for post‐translational modification, folding, organelle addressing, and secretion [3]. Cellular intrinsic or extrinsic factors can lead to an accumulation of unfolded or misfolded proteins within the ER [4]. Such alteration of proteostasis results in an ER stress that triggers an adaptive response named the unfolded protein response (UPR). The UPR aims to restore proteostasis within the organelle and involves three ER stress sensors that, once activated, limit *de novo* protein synthesis and favor protein folding and degradation [5]. These three ER stress sensors are the ER resident proteins activating transcription factor 6 alpha (ATF6α) [6], protein kinase RNA‐like endoplasmic reticulum kinase (PERK) [7] and the inositol‐requiring enzyme 1 (IRE1) [8].

## IRE1 signaling pathways

IRE1 exists in two isoforms, IRE1α and IRE1β. The former is ubiquitously expressed, whereas the latter is restricted to mucus‐secreting cells in the respiratory and gastrointestinal tract [9]. Here, we focus on IRE1α as it is the more extensively studied isoform and will refer to it as IRE1 hereafter. The cytosolic part of IRE1 comprises two catalytic domains: a kinase and an endoribonuclease (RNase) domain. Upon ER stress, IRE1 oligomerizes and trans‐autophosphorylates thanks to its kinase activity, thus enabling its RNase activity [10]. Through its RNase activity, IRE1 mainly regulates two signaling pathways: (i) the unconventional splicing of X‐box binding protein 1 (XBP1) mRNA, which involves cleavage of the XBP1 mRNA by IRE1 RNase domain and ligation by the tRNA ligase RNA 2′,3′‐cyclic phosphate and 5’‐OH ligase (RTCB), resulting in the formation of the transcription factor (TF) XBP1 spliced (XBP1s) able to modulate the expression of its target genes; and (ii) the regulated IRE1‐dependent decay (RIDD) of RNAs. In addition to its enzymatic activities, IRE1 has been shown to act as a scaffold through protein–protein interactions [11].

### The XBP1s pathway

XBP1s synthesis is not only dependent on IRE1 RNase activity but also relies on a multitude of factors that evolve along with proteostasis stress, such as translation regulation, transcript stability, or accessibility. The stability of XBP1s mRNA varies during the UPR and is regulated by a positive feedback loop arising from crosstalk between IRE1 and PERK signaling [12]. During the early phase of ER stress (within the first few hours), IRE1‐mediated splicing generates XBP1s mRNA, while PERK‐dependent phosphorylation of the eukaryotic initiation factor 2 α (eIF2α) inhibits its translation, allowing the accumulation of a ready‐to‐translate pool of transcripts. As the UPR progresses (after several hours), eIF2α dephosphorylation restores translation, thereby enabling XBP1s protein production. This recovery of translation accelerates XBP1s mRNA turnover, transiently increasing, but ultimately limiting XBP1s protein accumulation. Overall, this mechanism creates a temporal separation between XBP1s mRNA accumulation and protein synthesis [12]. This process is also controlled by the intensity and duration of ER stress. During the acute phase of ER stress, newly transcribed XBP1s mRNAs are synthesized with longer poly(A) tails, allowing them to escape translational repression [13]. Under chronic ER stress, these long poly(A)‐containing mRNAs undergo deadenylation, shortening their poly(A) tails and increasing their destabilization [13]. In *Drosophila*, Xbp1 mRNA is transiently sequestered in Ataxin‐2‐containing granules during early ER stress, where it is stabilized but translationally repressed. Subsequently, F‐box protein 42‐mediated degradation of Ataxin‐2 releases Xbp1 mRNA for translation, thereby controlling the timing of XBP1s production [14]. XBP1s is widely recognized as a pro‐adaptive factor across various pathological contexts, such as neurodegenerative diseases [15, 16] and cancers [17, 18, 19, 20]. It enhances protein folding, ER‐associated degradation, and other signaling pathways, thereby restoring ER proteostasis and supporting cell adaptation under stress conditions [21, 22]. Recent studies have also identified XBP1s as a transcriptional repressor that supports cell survival during acute ER stress by downregulating the expression of the p53 upregulation modulator of apoptosis (PUMA) [23], and promotes leukemia stem cell proliferation through repression of β‐catenin targets [24]. Therefore, XBP1s is a central element of the ER stress response, whose synthesis, regulation and impact remain to be fully unraveled.

### The RIDD pathway

Through the RIDD process, IRE1 directly cleaves messenger RNAs, ribosomal RNAs, and microRNAs in the cytosol. These RIDD targets contain a canonical CNG|CAGN motif within the loop of a RNA hairpin structure [25]. Nevertheless, some targets lacking such canonical motif have been described in a more promiscuous process called RIDD lacking endomotif (RIDDLE), revealing a broader range of RNAs susceptible to IRE1‐mediated degradation [26]. This process may be linked to the phosphorylation and oligomerization status of IRE1 [11]. However, the precise levels of phosphorylation or oligomerization required for XBP1 splicing, RIDD or RIDDLE activation remain highly debated, and the mechanisms underlying IRE1 oligomer formation are still poorly characterized. In addition, proteins interacting with IRE1 can modulate its RNAse activity [11]. This highlights that numerous levels of regulation modulate IRE1 activity and therefore the RIDD branch.

Given the involvement of IRE1 in the progression of several diseases, and the fact that its enzymatic activity can be pharmacologically targeted, it has raised considerable interest in medical research fields [27, 28]. However, the duality of IRE1 RNase activity, which on one hand favors gene expression via XBP1s; and on the other hand, reduces mRNA level through RIDD or RIDDLE; complicates the understanding of IRE1 role in disease progression. Indeed, while IRE1 activation primarily favors cell adaptation to stress, a failure to resolve proteostasis stress can result in IRE1 signaling‐induced cell death [29, 30]. How XBP1s and RIDD signaling pathways each weight toward the pro‐adaptive versus pro‐death phenotype is still poorly characterized [25]. In addition, the conditions favoring one or the other of IRE1 branches are still elusive. Currently, it is admitted that stress intensity and duration are key elements tilting the balance. Indeed, while a short and low intensity stress favors XBP1s signaling, a longer and more intense stress promotes the RIDD branch [25, 31]. Importantly, enhanced RIDD activity is exacerbated in XBP1 knock‐out dendritic cells [32, 33], B cells [34] and pancreatic β cells [35], highlighting the existence of a regulated feedback circuit between the branches of IRE1 signaling. Therefore, better understanding the dual and opposing signaling of this ER stress sensor in an integrated manner is necessary to improve our knowledge of IRE1 outcomes, which would greatly help harnessing IRE1 activities for therapeutic perspectives.

## Dual IRE1 targets (DIT): Key determinants of cell fate?

Recently, dual IRE1 targets (DIT), that are oppositely regulated by both XBP1s and RIDD signaling have been identified in our laboratory [31, 36]. We hypothesize that DIT could be key determinants of cell fate as decisive elements to tilt the pro‐adaptive versus pro‐death balance of the UPR. We define DIT as targets that are upregulated by XBP1s while being downregulated through RIDD or RIDDLE. Hence, DIT could prove highly interesting as targets whose fine regulation by IRE1 can favor cell adaptability or death, in response to an evolving environment.

## CD95 and UBE2D3 as DIT in cancer cells

### CD95

**Fig. 1:**
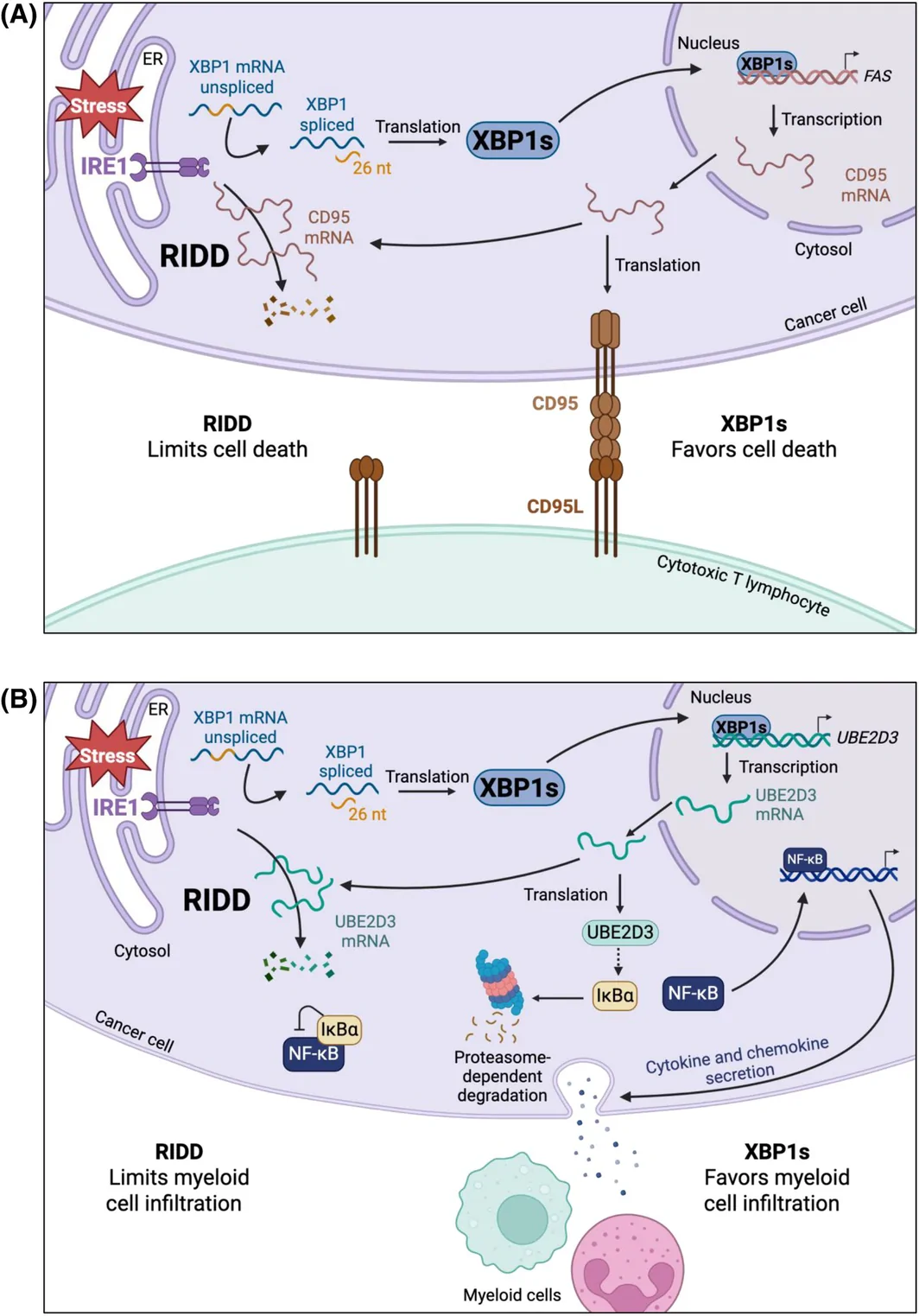
Dual IRE1 Targets (DIT) are central to cell adaptation and interaction with the microenvironment during ER stress. (A) CD95 is regulated dually by IRE1 activity. CD95 mRNA level is downregulated by RIDD, resulting in lower CD95 protein level, thus limiting CD95‐mediated extrinsic apoptosis induction. Within the other branch of IRE1 signaling, XBP1s upregulates CD95 transcripts, leading to a higher CD95 protein level at the cell surface. This sensitizes cancer cells to CD95L‐induced cell death. (B) The E2 ubiquitin‐conjugating enzyme UBE2D3 is also dually regulated by IRE1 signaling and contributes to the proteasome‐dependent degradation of IκBα, an inhibitor of the transcription factor NF‐κB. UBE2D3 RIDD degrades the UBE2D3 transcript, thus limiting NF‐κB activation, whereas XBP1s favors UBE2D3 expression, enabling NF‐κB activation and therefore allowing secretion of cytokine and chemokine‐attracting myeloid cells.

CD95 is a death receptor (DR) implicated in extrinsic apoptosis induction and various noncytotoxic outcomes, thereby impacting tumor progression [37]. In glioblastoma (GB) and triple‐negative breast cancer (TNBC) cells, we recently demonstrated that IRE1 dually controls CD95 transcript level and thereby, oppositely impacts cancer cell survival (Fig. 1A) [31]. We first observed that IRE1 cleaves CD95 mRNA *in vitro*. Moreover, in tumor cells, CD95 transcript and protein levels increase upon genetic or pharmacological inhibition of IRE1 RNase activity, under basal, intense or prolonged ER stress conditions, highlighting that inhibition of RIDD allows CD95 transcript and protein accumulation. Accordingly, genetic inhibition of IRE1 sensitizes GB cells to CD95 ligand (CD95L)‐induced caspase activation and cell death, thus showing that the RIDD‐induced reduction of CD95 transcript level impacts cell survival. As similar results were observed in both GB and TNBC cells, IRE1 effect on CD95 may be conserved between cancer types. On the contrary, XBP1s overexpression in GB or TNBC cells increases CD95 protein level and sensitizes them to CD95L‐induced cell death. Furthermore, *in vivo* pharmacological inhibition of IRE1 RNase using MKC‐8866 reduces CD95 protein level in hepatic cells and represses hepatotoxicity induced by a CD95 agonistic antibody. Hence IRE1, through XBP1s, promotes CD95 expression and CD95‐mediated cell death. Finally, by leveraging previously published RIDD and XBP1 gene signatures [20], we observed that CD95 mRNA level is elevated in TNBC and GB human tumors exhibiting low RIDD activity, whereas it is lower in XBP1s‐low human tumor samples. Taken together, this work highlights how IRE1 RNase signaling, by oppositely regulating a DR through both XBP1s and RIDD, is a determinant of GB and TNBC cell fate.

Interestingly, the expression of additional DRs (TRAIL‐R1/R2 and TNFR1) also seems to be correlated with IRE1 activity in TNBC and GB tumors, with a predominance of the XBP1s branch in TNBC tumors, while a dominance for the RIDD branch is observed in GB tumors. The predominant impact of the IRE1 branch controlling the expression of a given DIT could therefore vary depending on the stress intensity and duration, but also change between cell types or cellular context [31]. In addition, DRs may be regulated by UPR mediators besides IRE1. For instance, TRAIL‐R2/DR5 is upregulated by C/EBP homologous protein (CHOP) but downregulated by RIDD [38]. Hence, considering that the other UPR branches impact DR expression and signaling [37] and crosstalk with each other [39, 40, 41], an integrated analysis of DIT (including but not limited to DRs) expression in the context of each UPR branch activation is warranted.

### UBE2D3

Concomitantly to the identification of CD95 as a DIT, we identified the ubiquitin‐conjugating enzyme E2 D3 (UBE2D3) as a novel DIT (Fig. 1B) [36]. In GB, IRE1 is involved in myeloid cell infiltration (i.e., polymorphonuclear neutrophils, microglial cells, and macrophages) by controlling cytokine and chemokine secretion implicated in myeloid cell attraction. Inflammatory cytokine expression is often regulated by the TF nuclear factor‐kappa B (NF‐κB), whose transcript signature is positively associated with IRE1 and XBP1s and negatively associated with RIDD signature in GB specimens. Like NF‐κB, IRE1 and XBP1s signatures correlate with higher UBE2D3 transcript level. Notably, UBE2D3 participates in the degradation of the NF‐κB inhibitor alpha (IκBα), an inhibitor of NF‐κB, leading to the upregulation and secretion of cytokines and chemokines. We identified XBP1s binding sites in the promoter of the human and murine *UBE2D3* gene and confirmed their targeting by XBP1s using a gel shift assay. Additionally, stable repression of endogenous IRE1 RNase activity by overexpression of dominant negative form of IRE1 results in reduced UBE2D3 mRNA level, which is rescued upon XBP1s overexpression, thus supporting that XBP1s promotes *UBE2D3* gene expression. In addition, UBE2D3 mRNA was also identified as an *in vitro* RIDD target, although RIDD‐high signature does not correlate with changes in UBE2D3 transcript levels in GB tumors. UBE2D3 protein level decreases upon ER stress induction with thapsigargin (Tg) which is prevented by MKC‐8866, suggesting that IRE1 downregulates UBE2D3, likely through RIDD. Further, *in vivo* experiments demonstrated that UBE2D3 overexpression leads to increased activation of NF‐κB and infiltration of myeloid cells at the tumor site. On the other hand, UBE2D3 down‐regulation results in decreased tumor size and improves mice survival. This work demonstrates that IRE1 dual regulation of UBE2D3 modulates the NF‐κB pathway which, in turn, impacts cytokine and chemokine secretion, consequently affecting myeloid cell recruitment to the tumor site and therefore tumor progression. Hence, contrary effects of IRE1 signaling result in drastically opposite outcomes, regulating immune response and cancer progression, which could be exploited in future therapeutic perspective to either favor or limit inflammatory responses.

The IRE1‐dependent dual regulation of CD95 and UBE2D3 was discovered in a cancer context, specifically in GB where IRE1 is known to impact tumor development and aggressiveness [4, 42, 43, 44, 45]. Noteworthy, in GB, the XBP1 branch appears to be the dominant one for UBE2D3 regulation, while RIDD predominantly controls CD95, suggesting IRE1 branch sensitivity varies depending on each DIT. Because CD95 dual modulation by IRE1 was also observed in TNBC cells and given how common the influence of NF‐κB in tumor progression is among various cancer types, one cannot help but wonder whether UBE2D3 is a DIT target in additional cancers, and if so, whether the dominance of branches may change depending on cancer types, as mentioned for DRs earlier. Overall, cancer is a pathological context where modulating IRE1 to control consequences of its activities on cell fate could be highly promising as a therapeutic perspective.

## Other DIT identified in the literature are implicated in various pathological contexts

Here, we highlighted how DIT is shaping cell death and immune response in the specific context of cancer. Additionally, other targets of IRE1 have been independently identified as regulated by both XBP1s and RIDD in the literature. These targets are associated with various cellular pathways, such as protein homeostasis or lipid biosynthesis, and are implicated in diverse pathologies. We recapitulate DIT identified so far in Table 1.

**Table 1:** DIT identified in the literature. ActD, actinomycin D; CD95L, CD95 ligand; DN, dominant negative; OE, overexpression; Tg, thapsigargin; Tun, tunicamycin.

Symbol	IRE1 branch	Model	Stress condition	Cellular and molecular effects	Ref
BiP/GRP78	XBP1s	Th2 lymphocytes		XBP1s binds to BiP (*HSPA5*) promoter	[56]
	XBP1s	HeLa line	Tun	↗ Reporter expression and mRNA accumulation by XBP1s upon Tun	[57]
	RIDD	Rat insulinoma line INS‐1	Tg + ActD	↘ mRNA upon Tg + ActD	[30]
CD95	XBP1s	Human GB (U87) and TNBC (SUM159) lines; Mice hepatic cells	Tun, Tg CD95L MKC‐8866	↘ Protein *in vivo* after IRE1 inhibition ↘ Protein upon XBP1 knockdown ↗ Protein and sensitivity to CD95L‐induced cell death in XBP1s OE cells	[31]
	RIDD	Human GB (RADH85, RADH87, U87) and TNBC (SUM159) lines	Tun, Tg CD95L MKC‐8866	mRNA cleaved *in vitro* by IRE1 ↗ mRNA, total and membrane protein in IRE1 DN cells ↘ Total protein upon ER stress, reversed after IRE1 inhibition ↗ Protein and CD95L‐induced death sensitivity by MKC‐8866 ↗ CD95L‐induced death sensitivity in IRE1DN cells	[31]
DGAT2	XBP1s	Human liver cancer line HepG2		↘ Promoter activity, mRNA and protein after XBP1 silencing	[58]
	XBP1s	Mouse hepatocytes Control and XBP1 KO	Tun	XBP1s binds to DGAT2 promoter region ↘ mRNA in murine XBP1 KO hepatocytes ↗ mRNA in XBP1s OE WT and XBP1 KO primary hepatocytes	[59]
	RIDD	Human breast cancer lines (MDA‐MB‐231, MDA‐MB‐468, HCC1806, BT‐549)	Tun, Tg, +ActD, Brefeldin A MKC‐8866	mRNA cleaved *in vitro* by IRE1, impeded by MKC‐8866 or cleavage site mutation ↗ mRNA after IRE1 inhibition ↘ mRNA upon ER stress, prevented by MKC‐8866	[60]
UBE2D3	XBP1s	Human GB lines (RADH87, U87)		XBP1s binds UBE2D3 promoter ↘ mRNA in mutant or IRE1 DN cells, rescued by XBP1s OE	[36]
	RIDD	Human GB lines (RADH87, U87)	Tg MKC‐8866	mRNA cleaved *in vitro* by IRE1 ↘ Protein upon stress, rescued by MKC‐8866	[36]

### BiP/GRP78

The binding immunoglobulin protein or glucose‐related protein 78 (BiP), a chaperone implicated in restoring proteostasis, is a plausible DIT. Because BiP expression is strongly regulated by ATF6, another ER stress effector [46] the dual regulation of BiP interrogates on how IRE1 activity could mitigate the stress response. Indeed, the XBP1s branch would potentiate BiP upregulation by ATF6 while the RIDD branch would restrain it. In addition, BiP interaction with the UPR sensors impedes their activation, making it a regulator of the UPR. Hence, lowered BiP levels would favor UPR prolongation while higher BiP levels could help attenuate it. Thus, IRE1 dual activity might influence UPR continuation or termination. Moreover, BiP subcellular relocalization from the ER lumen to the plasma membrane has been described in the literature and mechanistically involves IRE1 activity [46], raising interrogation regarding what other proteins could be relocated in an IRE1‐dependent fashion. BiP is implicated in neurodegenerative and metabolic diseases, as well as cancer and viral infection where ER stress is often induced. How, in such context, a dual regulation of BiP by IRE1 may impact cell fate and disease progression remains to be fully investigated.

### DGAT2

DGAT2 (diacylglycerol O‐acyltransferase 2) is an enzyme that catalyzes the final step of triglyceride synthesis. DGAT1 and DGAT2 catalyze the same reaction despite variation in their sequence, substrate preferences, tissue, and subcellular localization [47, 48]. This suggests that IRE1 activity can have highly variable outcomes on lipid homeostasis depending on cell types as it will strongly impact cells with higher levels of DGAT2 compared to cells with higher levels of DGAT1. DGAT activity is implicated in metabolic diseases, nonalcoholic fatty liver diseases and cancer. Inhibition of DGAT2 hinders tumor progression in different cancers as it limits tumor cell proliferation, invasion and metastasis, reduces lipid droplet formation, and increases sensitivity to radiation [48]. This underlines that lipid metabolism is not only essential for the cell's energetic balance but also for other cellular processes, such as epithelial–mesenchymal transition. Hence, a better understanding of DGAT2 modulation is needed, thus investigating the dual regulation by IRE1 could prove valuable.

## Besides DIT—a dual regulation of whole pathways

Here, we describe how individual targets can be dually and oppositely regulated by IRE1. We coined the term DIT to describe discrete targets regulated by both XBP1s and RIDD activities. However, the impact of IRE1 can be broader, extending to the regulation of entire pathways whose fine‐tuning depends on its opposing signaling outputs. This is the case for insulin biosynthesis and secretion. Although insulin is not directly dually regulated by IRE1, several proteins involved in the hormone biosynthesis and secretion are upregulated by XBP1s (such as the translocon, signal recognition particle, or protein disulfide isomerase) [35, 49, 50], while others are downregulated by RIDD (such as carboxypeptidase E2 and protein convertase 1 and 2) [30, 51, 52 ]. Of note, the dual regulation of gene expression has been previously described within the XBP1 branch. Indeed, unspliced Xbp1/Hac1 has been initially described as a transcription repressor of genes associated with cell growth, cell division, and metabolism in yeast [53], whereas the spliced form of Xbp1/Hac1 activates transcription under ER stress [54, 55]; already highlighting an IRE1‐dependent opposing regulatory capacity evolutionarily conserved across eukaryotes.

## To DIT or not to DIT, that is the question

The interest in identifying DIT relies on the opposing nature of IRE1 signaling. Therefore, IRE1 targets that are dually regulated in a similar manner (i.e., downregulated by RIDD and repressed at the transcriptional level by XBP1s) should not be considered DIT. Additionally, the biological relevance of dual IRE1 regulation of DIT remains an open question and must be addressed to better define its scope and limitations. This will require not only further investigation of DIT themselves, but also a deeper understanding of IRE1 signaling and ER stress response. Does DIT regulation involve the simultaneous activation of both branches? Could XBP1s‐ and RIDD‐mediated regulation of DIT occur independently of one another? Under which conditions (i.e., stress duration, intensity and inductor) is DIT preferentially regulated by RIDD or XBP1s? How does the cellular context influence this regulatory preference? Furthermore, the definition of DIT is restricted to IRE1 targets. However, as mentioned above with TRAIL‐R2/DR5 or insulin, some targets are regulated by other UPR mediators in addition to IRE1. This further highlights the importance of considering regulated targets within the broader context of ER stress signaling and, consequently, of carefully accounting the crosstalk between UPR mediators.

## Conclusion

How IRE1 is a determinant of cell fate has long been described in the literature, where a plethora of examples show the role of this UPR effector in both cell survival and death across a variety of diseases. Hence, IRE1 is a potent actor whose activity could be harnessed for therapeutic purposes. However, a crucial understanding regarding IRE1 signaling mechanism is still lacking and many layers of regulation impacting the onset of each pathway are being unraveled.

Here, we have reviewed DIT, targets of IRE1 that are oppositely regulated through its two signaling branches XBP1s and RIDD. We believe that DIT, because of their dual nature, could be key components to orient the cellular outcomes controlled by IRE1 RNase activity. This is evidenced by the effect of IRE1 signaling on modulating cancer cell death and interaction with the immune system, which greatly impacts cancer progression. Investigating DIT would help to elucidate the bases of IRE1/XBP1s/RIDD signaling, as various aspects of the molecular mechanism are still poorly understood, especially how and what balances IRE1 signaling. This includes defining which combinations of criteria control IRE1 branch activation such as its oligomerization status, its interacting partners, the nature, duration and intensity of ER stress, as well as the cellular state and tissular context. Similarly, features of IRE1 targets, and therefore DIT, remain to be further studied, including but not limited to their sequences and structures, their DNA/RNA interacting partners, their subcellular localization and abundance, and their regulation by other UPR effectors. Though few DIT have been identified so far, there certainly will be others described in the near future. Upcoming research on IRE1 could provide crucial insights to shed light on proteostasis‐altered diseases which include several of our major health challenges such as metabolic diseases, cancers, and pathologies linked to aging, where DIT may become an integral part of these research fields.

## Author contributions

Conceptualization and supervision: EL and TA. Funding acquisition: EB, EL, and TA. Visualization and writing‐original draft: LL and EB. Visualization and writing‐review and editing: EB, EL, and TA.

## Conflicts of interest

The authors declare no conflict of interest.
